# Attitudes toward the use of complementary and alternative medicine in children with gastrointestinal symptoms, a multicenter survey study among parents and pediatricians

**DOI:** 10.1007/s00431-026-07058-3

**Published:** 2026-05-15

**Authors:** M.N. Bloem, D.F. Baaleman, I.J.N. Koppen, M.W. Bijlsma, A.M. Vlieger, J. Goede, F.B. Plötz, S.T.A Teklenburg-Roord, F. de Lorijn, M.A. Benninga

**Affiliations:** 1 Department of Pediatric Gastroenterology and Nutrition, Emma Children’s Hospital, Amsterdam University Medical Centre Location AMC, Amsterdam, The Netherlands; 2 Amsterdam Gastroenterology Endocrinology Metabolism Research Institute, Amsterdam UMC, Amsterdam, The Netherlands; 3 Amsterdam Reproduction and Development Research Institute, Amsterdam UMC, Amsterdam, The Netherlands; 4 Emma Children’s Hospital, Amsterdam University Medical Centre Location AMC, Amsterdam, The Netherlands; 5 Department of Pediatrics, St Antonius Hospital, Nieuwegein, The Netherlands; 6 Department of Pediatrics, Spaarne Gasthuis, Hoofddorp, Haarlem The Netherlands; 7 Department of Pediatrics, Tergooi MC, Blaricum, The Netherlands; 8 Department of Pediatrics, Isala Clinics, Zwolle, The Netherlands

**Keywords:** Disorders of gut-brain interaction (DGBI), Functional gastrointestinal disorders (FGID), Constipation, Abdominal pain, Infant colic, Reflux, Integrative therapies

## Abstract

Disorders of gut-brain interaction (DGBI) are common in children and often persist despite conventional treatments, leading families to seek complementary and alternative medicine (CAM). However, evidence for CAM in pediatric DGBI is scarce. This multicenter survey study assessed parents’ and pediatricians’ experiences with and attitudes toward CAM for children with gastrointestinal symptoms. Parents and pediatricians of children (0–18 years) with gastroesophageal reflux disease (GERD) and DGBI (infant colic (IC), functional abdominal pain, and functional constipation) from six Dutch hospitals were included. GERD diagnosis was based on international guidelines, DGBI on Rome IV criteria. Parental surveys addressed child health, medication, (reasons for) CAM use, and attitudes toward CAM research. Pediatricians’ surveys covered experiences and attitudes toward CAM for pediatric DGBI. A total of 677 parents (71%) and 76 pediatricians (79%) responded. Most patients had functional constipation (45%) or abdominal pain (41%), with 8% IC and 6% GERD. Seventy-one percent had symptoms for over a year; 58% used medication, and 49% reported side effects. Overall, 42% of patients used CAM, mainly manual therapies, homeopathy, and natural remedies. Predictors of CAM use included IC, longer symptom duration, higher household income, and positive parental CAM experience. Among pediatricians, 53% recommended CAM. Pediatricians with over 15 years’ experience were less likely to recommend CAM. Sixty-five percent of parents were willing to participate in CAM research.

*Conclusion*: CAM use is common among children with DGBI or GERD, and over half of pediatricians have recommended CAM, underscoring the need for efficacy studies of frequently used CAM.

**Table:** 

**What is Known:** •* Complementary and alternative medicine (CAM) is commonly used among pediatric patients with gastrointestinal symptoms but often excluded in evidence-based guidelines due to insufficient evidence.* *• Previous research has examined perspectives of patients and families, with limited insight into pediatricians’ recommendations.*	
**What is New:** •* In Dutch hospitals, 42% of children with infant colic, reflux, abdominal pain, or constipation used CAM. Most pediatricians (52.6%) recommended some CAM to these patients.* *• Predictors for CAM use include infant colic, longer symptom duration, positive parental CAM experience, and higher income; pediatricians with > 15 years’ experience were less likely to recommend CAM. Parental interest in CAM research is high (65%).*

**Graphical Abstract:**

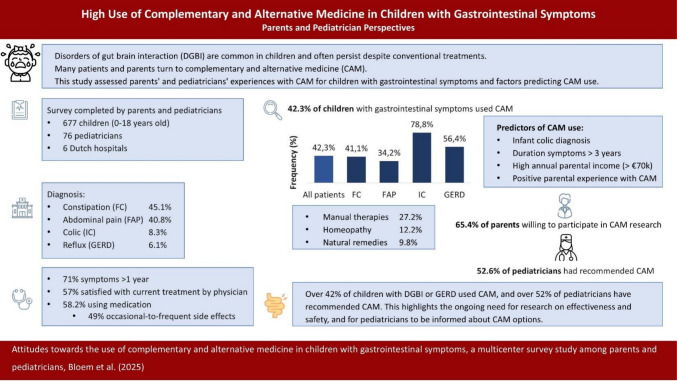

**Supplementary Information:**

The online version contains supplementary material available at 10.1007/s00431-026-07058-3.

## Introduction

Disorders of gut-brain interaction (DGBI) are common in children of all ages, affecting around one in four children [1, 2]. These conditions are bothersome and have a negative impact on children’s quality of life [2–4]. A large proportion of these children remains symptomatic despite conventional treatment [5–7]. Not surprisingly, one study reported that about 40% of parents of pediatric gastroenterology patients use complementary and alternative medicine (CAM) for their child [8]. Since CAM is being applied in clinical practice, there is a need not only to investigate the potential positive effects of these treatments but also to investigate possible adverse effects [9].

The Cochrane collaboration defined CAM as “a broad domain of healing resources that encompasses all health systems, modalities, and practices and their accompanying theories and beliefs, other than those intrinsic to the politically dominant health system of a particular society or culture in a given historical period” [10]. They have provided a list of CAM modalities including over 50 treatments and a footnote stating that depending upon the condition being treated, some treatments may be considered standard Western allopathic medicine [10]. One example is hypnotherapy to treat children with functional abdominal pain, which in recent years moved from the field of CAM to conventional medicine; currently, this is considered one of the most effective treatment options for children with abdominal pain–related DGBI [11, 12].

Among CAM modalities utilized in pediatric populations, a wide range of treatments have been explored for gastrointestinal symptoms, including but not limited to acupuncture, herbal medicine, and manual therapies such as osteopathy, chiropractic care, and massage. These approaches are rooted in various traditional and integrative medical systems, each with distinct philosophies and mechanisms of action. However, the evidence base for many CAM treatments in children with DGBI remains limited, and the safety profiles, feasibility, and acceptance of these modalities in pediatric clinical practice are not yet fully understood [13].

Therefore, further research is needed to evaluate both potential benefits and risks associated with CAM use in children with gastrointestinal disorders. The objective of this study was to examine experiences with and attitudes toward CAM in children with gastrointestinal symptoms among patients, parents, and healthcare providers, focusing on four most common pediatric gastrointestinal diagnoses: gastroesophageal reflux disease (GERD), infant colic (IC), functional constipation (FC), and functional abdominal pain (FAP) [14, 15].

## Methods

### Ethics statement

The study protocol was conducted in accordance with the Declaration of Helsinki and approved by the Ethics Committee of the Amsterdam University Medical Centers.

### Study design

This multicenter, cross-sectional survey study was conducted at six hospitals in the Netherlands, including one academic and five general hospitals. Surveys were collected between September 2022 and November 2024.

### Participants

A total of 953 patients and 96 general pediatricians were invited to participate. Eligible participants included parents or legal guardians of children (inpatients and outpatients, aged 0–18 years) who had been diagnosed with one or more of the following four common gastrointestinal disorders, GERD (diagnosed according to the ESPGHAN/NASPGHAN guidelines) [16] and three DGBI diagnosed according to the Rome IV criteria: IC, FC, and FAP (including irritable bowel syndrome and functional abdominal pain—not otherwise specified, Appendix 1) [14, 15]. These conditions were selected due to their high prevalence and sometimes limited treatment options [17–19]. Parents or guardians with insufficient Dutch language proficiency were excluded.

### Recruitment and consent

Parents or caregivers of eligible children were informed about the study by the treating pediatrician, who documented verbal consent and submitted the child’s diagnosis and parent’s email via Castor EDC. The investigator then sent an invitation for the online questionnaire, completed at home. Twenty €10 gift cards were randomly awarded as incentives. All participants provided informed consent. General pediatricians were invited by email with a link to the anonymous Castor questionnaire, distributed by the departmental secretary, with no incentive provided.

### Questionnaires

The self-developed parental questionnaire incorporated items from previous studies on attitudes toward CAM [8], the Acupuncture Expectancy Scale [20], previous acupuncture use studies [21, 22], and validated instruments such as the Beliefs about Medicine Questionnaire (BMQ) [23]. The survey assessed demographics, previous treatments for GERD or DGBI, satisfaction with conventional treatments, and the occurrence of adverse drug events as reported by parents. The pediatrician questionnaire assessed experience, attitudes, and beliefs regarding CAM. All responses were collected anonymously.

### Definition of CAM

Parents were provided with a list of commonly recognized therapies, including hypnotherapy, (electro)acupuncture, herbal medicine, natural remedies, manual therapies (i.e., osteopathy, chiropractic, massage), homeopathy, and other alternative therapies, as identified in the literature (Appendix) [8, 24]. All participants were explicitly asked about hypnotherapy use (participants) and its recommendation (pediatricians); hypnotherapy was classified as CAM only when used or recommended for the treatment of GERD or FC, when hypnotherapy was used or recommended for FAP, it was excluded from the analysis, as it is part of the current guidelines for children with FAP [11, 12].

### Statistical analysis

Data were collected in Castor EDC and analyzed using SPSS version 28 (IBM Corp., Armonk, NY) and R version 4.4.3 (R Core Team, Vienna, Austria). Descriptive statistics were presented as percentages, mean (standard deviation), or median (interquartile range or range), depending on data distribution. Normality was assessed using the Shapiro–Wilk test and visual inspection of histograms. Group comparisons for continuous variables were performed using independent *t*-tests or one-way ANOVA, with Levene’s test for homogeneity of variance where appropriate. Categorical variables were compared using chi-square tests or Fisher’s exact tests when expected cell counts were low. Univariate and multivariate logistic regression analyses were conducted to identify predictors of CAM use and CAM recommendation, with odds ratios (ORs) and 95% confidence intervals (CIs) reported. As a sensitivity analysis, we repeated the multivariate logistic regression including a variable indicating whether the mother was the survey respondent (compared to the father as a respondent reference group), to assess potential respondent bias. Parental attitudes toward medicines were assessed using the BMQ subscales: harm, overuse, necessity, concern, and difference scores [23]. Analyses were performed on available cases; missing data were not imputed. Statistical significance was set at *p* < 0.05.

## Results

### Patient and parent characteristics

Of 953 invited patients, 677 (71%) completed the questionnaire (median age of 9.4 years, 52% female). Most common diagnoses were FC (45.1%) and FAP (40.8%), followed by IC (8.3%) and GERD (6.1%). Satisfaction with pediatric care at time of survey completion was very high in 36.8% of parents and low in 8.1% (Table 1). Most respondents were mothers (89%), with fathers more frequently represented among non-CAM users (13.8% vs. 7.9%, *p* = 0.024). Most parents were highly educated (57%). High household income was more common among CAM users (35.7% vs. 27.8%, *p* = 0.036). A personal prior positive experience with CAM was reported by 22.3% of parents. Other demographic variables were similar between groups (Table 2).

**Table 1:** Patients’ demographics and disease data

Characteristic	Prevalence, *n* (%)
Age (years) (median/IQR/ranges)	9.4 (8.8; 0.1–18.9)
Patient’s gender (*N* = 673)	
Female	347 (51.6)
Male	323 (48)
Rather not to say	3 (0.4)
Hospital centers (*N* = 677)	
Academic hospital (1 center)	408 (60.3)
General hospitals (5 centers)	269 (39.7)
Type of diagnosis (*N* = 677)	
FC	305 (45.1)
FAP	276 (40.8)
IC	56 (8.3)
GERD	41 (6.1)
Ethnicity (*N* = 655)	
Caucasian	384 (58.6)
Not specified/not disclosed	125 (19.1)
Arabic	37 (5.7)
Other/mixed^*^	35 (5.3)
Asian	30 (4.6)
Latin American	29 (4.4)
African	15 (2.3)
Comorbidities present^**^ (*N* = 655)	140 (21.4)
Duration of symptoms (*N* = 655)	
> 1 year	504 (76.9)
Months	148 (22.6)
Weeks	3 (0.5)
Median of reported symptoms in years (IQR; range)	3.0 (5.0; 0.1–17.0 years)
Current medication use (*N* = 655)	382 (58.3)
Side effects among medication users (*N* = 382)	
None	194 (50.8)
Rare	92 (24.1)
Occasional or frequent	96 (25.1)
Treatment effectiveness (parental assessment) (*N* = 655)^***^	
Somewhat or very effective	342 (52.2)
Limited or not effective	185 (28.2)
Not yet started	128 (19.5)
Satisfaction with pediatrician’s treatment (parental assessment)	
High	241 (36.8)
Moderate	129 (19.7)
Neutral	121 (18.5)
Low	53 (8.1)
Not yet received treatment	111 (16.9)

Response rates varied per question

^*^Ethnicity was self-reported by parents. The category “other/mixed” includes children whose parents reported multiple ethnic backgrounds or whose ethnicity did not fit into the predefined categories

^**^Most common comorbidities mentioned included atopic disorders (25.0%), autism spectrum disorders and AD(H)D (18.6%), chromosomal deletions (6.4%), and other comorbidities, including cardiological, hematological, oncological, and endocrinological disorders

^***^Treatment effectiveness reflects parental assessment of symptom improvement. Satisfaction with pediatrician’s treatment indicates overall satisfaction with care and communication

**Table 2:** Parents’ demographics

Parent characteristics	Never used CAM for their child *n* (%) (*n* = 378)	Used CAM for their child *n* (%) (*n* = 277)	Total prevalence *n* (%) (*n* = 655)	*p* value
Age (years) (mean, SD)	41.4 (7.7)	40.5 (8.0)	41.0 (7.8)	0.337
Relationship with child				**0.024**
Mother	325 (86.0)	255 (92.1)	580 (88.5)	
Father	52 (13.8)	22 (7.9)	74 (11.3)	
Other caregiver (female)	1 (0.3)	0 (0.0)	1 (0.2)	
Relationship with other partner				0.165
Cohabiting	77 (20.4)	71 (25.6)	148 (22.6)	
Married	231 (61.1)	163 (58.8)	394 (60.2)	
Divorced	40 (10.6)	31 (11.2)	71 (10.8)	
Widow state	2 (0.5)	1 (0.4)	3 (0.5)	
No other parent/caretaker involved	20 (5.3)	5 (1.8)	25 (3.8)	
Others, namely^*^	8 (2.1)	6 (2.2)	14 (2.1)	
Highest education obtained				0.073
Primary school	0 (0.0)	1 (0.4)	1 (0.2)	
Secondary vocational school (MBO)	23 (6.1)	15 (5.4)	38 (5.8)	
General secondary school (HAVO/VWO)	149 (39.4)	85 (30.7)	234 (35.7)	
University	200 (52.9)	174 (62.8)	374 (57.1)	
Others, namely	1 (0.3)	0 (0.0)	1 (0.2)	
Not specified	5 (1.3)	2 (0.7)	7 (1.1)	
Income				**0.036**
< €24.000	24 (6.3)	9 (3.2)	33 (5.0)	
€24.000–€35.000	27 (7.1)	17 (6.1)	44 (6.7)	
€35.000–€70.000	110 (29.1)	90 (32.5)	200 (30.5)	
> €70.000	105 (27.8)	99 (35.7)	204 (31.1)	
I’d rather not to say Parental experience with CAM No or undisclosed experience Positive experience	112 (29.6) 326 (86.2) 52 (13.8)	62 (22.4) 183 (66.1) 94 (33.9)	174 (26.6) 509 (77.7) 146 (22.3)	** < 0.0001**

Statistical comparisons were performed using the chi-square test for categorical variables, Fisher’s exact test when expected cell counts were < 5, and the Wilcoxon rank-sum test for continuous variables. Values in bold indicate statistical significance at *p < 0.05*.

^*^Registered partnership and not cohabiting

### Predictors of CAM use

Univariate logistic regression identified several significant predictors of CAM use (Table 3); gender, parental education, lack of perceived effectiveness of treatment by the pediatrician, and medication side effects were not significantly associated with CAM use. In multivariate analysis, independent predictors of CAM use were a diagnosis of IC, gastrointestinal symptoms for more than 3 years, high household income, and positive parental experience with CAM (Table 3). Adjusting for respondent type did not change the findings (Appendix 2).

**Table 3:** Variables associated with CAM use*

Variable	Univariate analysis OR (95% CI)	Multivariate analysis OR (95% CI)
Age (younger)	0.97 (0.94–1.00)	NS
Gender (female)	NS	NS
IC diagnosis	5.80 (3.02–12.06)	3.05 (1.04–8.99)
FAP diagnosis	0.58 (0.42–0.80)	NS
Parental education (high)	NS	NS
High household income family (> €70.000)	1.31 (0.91–1.89)	1.90 (1.02–3.53)
No perceived effect of treatment by pediatrician	0.88 (0.48–1.62)	NS
Gastrointestinal symptoms > 3 years	1.32 (0.97–1.80)	2.60 (1.30–5.20)
Parental experience with CAM (good)	3.22 (2.18–4.76)	3.28 (1.70–6.34)

*NS* not significant

^*^If patients reported having used hypnotherapy for FAP specifically, this was excluded from the analysis, as hypnotherapy is not considered CAM for FAP and is prescribed according to current clinical guidelines in this cohort

### CAM use in DGBI patients

Overall, 42.3% of children reported having used at least one CAM, mainly manual therapies (27.2%), homeopathy (12.2%), and natural remedies (9.8%, Appendix 3). CAM use was highest among patients with IC (78.8%). Also after excluding IC patients in the prevalence analysis due to IC being a predictor of CAM use, overall prevalence of CAM use remained high (39.0%). Among GERD patients, 56.4% used CAM, most commonly manual therapies, homeopathy, and hypnotherapy. Among FAP patients, 34.2% used CAM, mainly manual therapies and homeopathy; 20.7% reported using hypnotherapy, but hypnotherapy for FAP was not included in the reported prevalence of CAM use of FAP patients (Appendix 4). In IC, the majority (71.2%) used manual therapies such as osteopathy. In FC, 41.1% of children with FC used CAM.

### Perceived effectiveness and parental motivations for CAM use 

Most parents rated CAM therapies as somewhat effective (23–60%), with a notable proportion finding them not effective (10–64.3%) or unsure (6.7–21.7%). Common reasons for choosing CAM included wishing to cure the condition (82%), a desire for more control or involvement in their child’s health (46.2%), a preference to use less medication (42.2%), and advice from friends or family (37.2%). Less frequent motivations included disappointment with conventional treatment, a preference for natural therapies, information from media, long waitlists for pediatric referrals, and a wish to combine conventional and alternative care. Among parents who reported no CAM use for their child at the time of the survey, 71.2% reported considering CAM if recommended by their treating pediatrician, while only 15.9% stated they had no reason to ever consider CAM (further elaborated in Appendix 5).

### Parental investment and research willingness in CAM

Mean reported CAM costs over the previous year were €282.67 (SD €451.66), with a median of €200 (range €0–5000). Nearly two-thirds (65.4%) of parents expressed interest in participating in future CAM research for their child’s symptoms.

### CAM use only weakly associated with parental beliefs about medicines

Overall, 10.7% of parents perceived medicines as harmful, 33.1% believed doctors overprescribe, 50.1% strongly endorsed the necessity of their child’s medicines, and 17.9% expressed concerns about potential side effects. CAM users scored slightly higher than nonusers on BMQ subscales [23] indicating greater skepticism and concerns regarding medicines, but mean differences were small (harm 10.0 vs. 9.6; overuse 11.8 vs. 11.0; Appendix 6). No differences were observed for overall BMQ difference scores, suggesting similar beliefs about medicines between groups. Among respondents using medication, 66.4% of CAM users felt their child’s health depended on medicines (vs. 36.2% of nonusers), and 53.9% worried about their use (vs. 14.1% of non-CAM users).

### Pediatrician characteristics and attitude toward CAM

Among 76 pediatricians (response rate 79.2%), 84.4% were general pediatricians working at general hospitals (92.2%), with over half (51.3%) having more than 15 years of experience and the majority being Caucasian (96.1%, Appendix 7). Forty (52.6%) reported recommending at least one CAM therapy for children with DGBI, most commonly hypnotherapy (38.2%—this number excludes patients with FAP) and manual therapies (28.9%). For FC, 32.9% had recommended CAM (mainly hypnotherapy (27.4%) and homeopathy (4.1%)); for FAP, 15.1% had recommended CAM (mainly homeopathy (5.5%) and manual therapies (4.1%)). If hypnotherapy for FAP is included in the analyses, 98.6% of pediatricians reported recommending it (Appendix 4). For IC, 26.1% had recommended CAM (mainly manual therapies (21.7%) and natural remedies (2.9%)), and for GERD, 18.3% had recommended CAM (mainly hypnotherapy (12.7%) and manual therapies (4.2%)).

### Predictors of CAM recommendation by pediatricians

In univariate analysis, pediatricians with more than 15 years of work experience were less likely to recommend CAM compared to those with 0–5 years of experience (OR 0.28, 95% CI 0.08–0.87, *p* = 0.036). No significant association was found for work experience of 6–10 years or 11–15 years, nor for other pediatrician characteristics, including specialization, hospital type (general vs. academic), province, and ethnicity.

## Discussion

In this Dutch multicenter survey, 42% of children with GERD or DGBI reported CAM use, mainly manual therapies, homeopathy, and natural remedies, with highest use in infant colic (79%). Over half of pediatricians recommended CAM.

The prevalence of CAM use in our study aligns with previous findings in Dutch children with DGBI (37.6%) and oncological diagnoses (42%) [8, 24]. Internationally, rates vary: in the USA, CAM use was reported in 96% of children with FAPD and 25% with FC [25, 26]. Turkish studies report even higher rates, with over half of parents using herbal supplements for DGBI, often without medical advice, and overall CAM use ranging from 55% for gastrointestinal symptoms to 97% for any symptoms [27, 28].

In our cohort, manual therapies and homeopathy were most frequently used CAM, with osteopathy being predominant among infants with colic. Systematic and scoping reviews of manual therapies for IC report mixed results, generally indicating no to moderate benefits, low-quality evidence, and methodological variability [25–27]. Although small benefits may exist, it remains unclear whether these are meaningful to parents. Despite high CAM use in our cohort, only a minority of parents rated these therapies as very effective, with most reporting moderate benefit. This subjective assessment aligns with a German study in children with FC, where parental interest in CAM remained high despite limited objective improvements [28].

Parents’ main reasons for CAM use, including improving health, reducing side effects, managing symptoms, psychological support, and last resort attempts, align with findings in Turkish and Dutch studies, which highlight symptom relief, dissatisfaction with conventional treatments, preference for natural therapies, and positive parental experiences [8, 29, 30]. A recent systematic review of 213 publications worldwide on CAM use in general identified expectations of benefit, dissatisfaction with conventional medicine, and perceived safety as the most common reasons [31]. Internal health locus of control (how individuals believe they can influence their own health) was more influential in Western countries, while social networks and tradition played a larger role in Asian and African regions [31].

Predictors for CAM use in our cohort included a diagnosis of infant colic, a positive parental experience with CAM, gastrointestinal symptoms lasting more than 3 years, and higher household income. These findings showed both similarities and differences with previous studies [8, 28, 32–38]. While mothers were more often respondents, sensitivity analysis indicated respondent bias was unlikely. The relationship between parental income and CAM use varies: Some studies, like those on children with juvenile idiopathic arthritis, link higher income to increased CAM use, while a study from New Zealand found no such association, suggesting CAM’s wider accessibility across socioeconomic groups [32, 33, 39]. In another study where high household income was not a significant predictor of CAM use, higher parental education emerged as a predictor, which may correlate with higher income [36, 38, 40].

While ineffectiveness of conventional treatment has been linked to CAM use in other studies, it was not a significant predictor in our analysis, nor were negative beliefs about medicines [8]. This suggests that CAM use may represent an additional step rather than a response to dissatisfaction with conventional treatment, as adherence to conventional treatments, as shown in another study, was similar in CAM users and nonusers [34]. Additionally, the association between longer symptom duration (> 3 years) and CAM use in our cohort may have overshadowed perceived ineffectiveness. This aligns with findings in adult IBD, but contrasts with a Dutch pediatric study (with a shorter duration cut-off of ≤ 3 months) which found no association between symptom duration and CAM use [8, 41].

A positive parental experience with CAM independently predicted CAM use in our cohort, consistent with previous studies in childhood constipation, IBD, and epilepsy [28, 34, 35]. An IC diagnosis predicted CAM use, likely reflecting limited effective conventional treatment options for IC, more so than for GERD, FC, and FAP. Current management primarily consists of reassurance, with occasional hospital admission, mainly to alleviate parental stress.

Previous Dutch and American surveys show pediatricians’ attitudes toward CAM are cautious but increasingly open, with referral rates around 30% in the Netherlands and similar patterns in the USA [36, 37, 42]. In our current survey targeting pediatricians treating GERD and DGBI, over 50% reported having recommended some form of CAM, a notably higher proportion than previously reported, although our sample was smaller but had a higher response rate. This difference suggests CAM may be more frequently considered in functional gastrointestinal symptoms, possibly reflecting the chronic and refractory nature of these conditions and the demand for integrative approaches in this group, or a shift in attitudes over time, as the previous Dutch survey was conducted in 2011 [42]. Pediatricians with over 15 years’ experience were less likely to recommend CAM, consistent with previous findings that greater clinical experience may be associated with more cautious attitudes toward CAM [43] and potentially reflecting recent developments in pediatric care and evolving cultural attitudes toward CAM.

Clinicians should be aware of the high prevalence of CAM use and engage in open discussions with families about its potential benefits, risks, and interactions with conventional therapies [28]. Most parents in our study held positive attitudes toward conventional medicines, with low concern about harm or overuse and a strong belief in their necessity. While CAM users showed slightly more skepticism and concern, differences in BMQ subscale scores were small. Notably, CAM users were more likely to both recognize the necessity of medication and express worry about its use, suggesting a nuanced attitude rather than a major influence of general medicine beliefs on CAM use.

Among parents who reported no CAM use for their child, only 15.9% stated they would never consider any CAM. The majority expressed openness to CAM under certain circumstances, particularly if recommended by a healthcare provider or for reasons such as improving general health or taking a proactive approach, highlighting that attitudes toward CAM are multifaceted.

This is the first study to systematically explore CAM use among Dutch pediatric patients with gastrointestinal symptoms, incorporating perspectives of both parents and pediatricians. The large, diverse, multicenter cohort, from both academic and general hospitals, enhances generalizability. The response rate among pediatricians (79.1%) is much higher than some previous pediatrician survey studies [36, 37, 42], supporting representativeness. Use of validated instruments, such as the BMQ, strengthens assessment of parental attitudes.

Limitations include potential nonresponse and recall bias, as parents with strong opinions may be more likely to participate and retrospective reporting may be inaccurate, and ethnic (predominantly Caucasian) and socioeconomic selection bias, due to 57% of parents being highly educated, which is substantially higher than 37% in the general Dutch population (Central Bureau of Statistics, 2024). Diagnostic group distribution across centers, with FC overrepresented in academic and FAP, IC, and GERD in general centers, as well as small IC and GERD groups may limit power to detect differences. Diagnoses were confirmed by the treating pediatrician at the time of recruitment, although no systematic chart review was performed which may have introduced some diagnostic misclassifications. Hospital-based recruitment may reduce generalizability to primary care, where CAM use may be even higher. We also did not assess pediatricians’ personal CAM use, a known predictor of CAM recommendation [42].

Differences in CAM definitions and exclusion of certain therapies may underestimate prevalence and limit comparability. Our study did not explicitly include certain therapies, such as vitamins, Schuessler tissue salts, or Bach flower remedies, which were included in other studies [28]. Standardization of CAM definitions is complicated by cultural and healthcare system differences, that is, acupuncture and TCM are mainstream in China, Ayurveda is recognized in India, and whether some Western countries include certain therapies in their hospital settings varies widely. In our cohort, hypnotherapy was classified as CAM only when used or recommended for treatment of symptoms of GERD or FC, not FAP, as it is part of current clinical guidelines for these children.

While research into the efficacy of specific CAM therapies is increasing, robust pediatric data remain limited. For example, massage therapy may benefit constipation in children with cerebral palsy [44], and acupuncture shows efficacy for adult DGBI [45, 46], but high-quality pediatric studies are needed. Safety of CAM, particularly for modalities like osteopathy, chiropractic, herbal treatments, and acupuncture, among others, was not systematically assessed in our study and also warrants further prospective investigation. Notably, 65.2% of parents in our study expressed willingness to participate in future CAM research, which is even higher than previously reported in parents with children with oncological disorders [24] underscoring the need and potential for future research on both efficacy and safety of CAM.

## Conclusion

Parents commonly report CAM use (42%) for their children with gastrointestinal symptoms, with 65% willing to participate in future CAM research, reflecting strong interest in these therapies. Additionally, over half of pediatricians have recommended CAM, indicating a generally positive attitude among clinicians. Given the high prevalence of CAM use, parental engagement, and supportive stance of pediatricians, high-quality studies on efficacy of frequently used CAM are urgently needed.

## Supplementary Information

Below is the link to the electronic supplementary material.

ESM 1 (DOCX 62.7 KB)

## Data Availability

Data will be available upon reasonable request.

## Abbreviations

DGBI: Disorders of gut-brain interaction
CAM: Complementary and alternative medicine
FAP: Functional abdominal pain
FC: Functional constipation
GERD: Gastroesophageal reflux disease
IC: Infant colic
TCM: Traditional Chinese medicine
