# Supplementary material for: Attitudes toward the use of complementary and alternative medicine in children with gastrointestinal symptoms, a multicenter survey study among parents and pediatricians
Source: Eur J Pediatr. 2026 May 15;185(6):398. doi: 10.1007/s00431-026-07058-3 (PMC13179196; doi:10.1007/s00431-026-07058-3)
Supplement: Supplementary file 1 — (DOCX 62.7 KB) [file 431_2026_7058_MOESM1_ESM.docx]

**Appendix**

**Appendix 1. Diagnostic criteria for functional constipation, functional abdominal pain, infant colic and gastroesophageal reflux**

1. **Functional constipation**

| Rome IV criteria for functional constipation in children and adolescents ^1,2^ must include 2 or more of the following occurring at least once per week for a minimum of 1 month with insufficient criteria for a diagnosis of irritable bowel syndrome a:   1. 2 or fewer defecations in the toilet per week in a child of a developmental age of at least 4 years 2. At least 1 episode of fecal incontinence per week 3. History of retentive posturing or excessive volitional stool retention 4. History of painful or hard bowel movements 5. Presence of a large fecal mass in the rectum 6. History of large diameter stools that can obstruct the toilet   After appropriate evaluation, the symptoms cannot be fully explained by another medical condition. |
| --- |
|  |

1. **Functional abdominal pain (including irritable bowel syndrome and functional abdominal pain – not otherwise specified)**

Rome IV diagnostic criteria* for childhood functional abdominal pain ^1,2^ must include all of the following:

1. Episodic or continuous abdominal pain

2. Insufficient criteria for other FGIDs

3. No evidence of an inflammatory, anatomic, metabolic, or neoplastic process that explains

the subject’s symptoms

**Criteria fulfilled at least once per week for at least 2 months before diagnosis*

^a^ Rome IV diagnostic criteria* for irritable bowel syndrome (IBS) in children and adolescents must include all of the following:
1. Abdominal pain at least 4 days per month associated with one or more of the following:
 a. Related to defecation
 b. A change in frequency of stool
 c. A change in form (appearance) of stool
2. In children with constipation, the pain does not resolve with resolution of the constipation
(children in whom the pain resolves have functional constipation, not irritable bowel syndrome)
3. After appropriate evaluation, the symptoms cannot be fully explained by another medical condition

** Criteria fulfilled for at least 2 months before diagnosis*

1. **Infant colic**

Rome IV criteria for infant colic ^1,2^ must include all of the following in infants from birth to 4 months of age:

1. Paroxysms of irritability, fussing, or crying that start and stop without obvious cause

2. Episodes lasting 3 or more hours per day and occurring at least 3 days per week for at least 1 week

3. No failure to thrive.

1. **Gastroesophageal reflux disease**

Gastroesophageal reflux disease is defined as the passage of gastric contents into the esophagus with or without regurgitation and vomiting which leads to troublesome^*^ symptoms that affect daily functioning and/or complications ^3^

* Reported symptoms of infant GERD vary widely and may include excessive crying, back arching, regurgitation and irritability. Many of these symptoms, however, occur in all babies with or without GERD, making a definitive diagnosis challenging. Therefore, the degree of concern of parents is often the factor driving the need for a diagnosis. For older children (particularly those older than the age of 8) and adolescents who can communicate more effectively, typical symptoms such as heartburn and regurgitation mimic those seen in adults with GERD.^3^

**References**

^1^ Benninga MA, Nurko S, Faure C, Hyman PE, St. James Roberts I, Schechter NL. Childhood functional gastrointestinal disorders: neonate/toddler. Gastroenterology. 2016;150(6):1443-1455.

^2^ Hyams JS, Di Lorenzo C, Saps M, Shulman RJ, Staiano A, van Tilburg M. Childhood functional gastrointestinal disorders: child/adolescent. Gastroenterology. 2016;150(6):1456-1468.

^3^ Rosen R, Vandenplas Y, Singendonk M, et al. Pediatric Gastroesophageal Reflux Clinical Practice Guidelines: Joint Recommendations of the North American Society for Pediatric Gastroenterology, Hepatology, and Nutrition and the European Society for Pediatric Gastroenterology, Hepatology, and Nutrition. Journal of pediatric gastroenterology and nutrition. 2018;66(3):516- 554.

**Appendix 2. Predictors of CAM Recommendation and CAM Use, including the mother as the respondent**

In univariate analysis, significant predictors of CAM use included diagnosis of IC (OR: 5.80, 95% CI: 3.02–12.06), diagnosis of FAP as a negative predictor (OR: 0.58, 95% CI: 0.42–0.80), younger age (OR: 0.97, 95% CI: 0.94–1.00), and the mother being the respondent of the survey (OR: 1.89, 95% CI: 1.13–3.25). In multivariate analysis, independent predictors of CAM use were diagnosis of IC (OR: 3.66, 95% CI: 1.29–11.42), high household income (OR: 2.23, 95% CI: 1.19–4.26), gastrointestinal symptoms for more than three years (OR: 2.15, 95% CI: 1.10–4.27), and the mother being the respondent of the survey (OR: 5.29, 95% CI: 1.94–17.24). Other variables, including gender, parental education, and perceived effectiveness of treatment, were not significant in the multivariate model.

| Table 2 | Variables associated with CAM use* | | |
| --- | --- | --- | --- |
| Variable | | **Univariate Analysis,**  **OR (95% CI)** | **Multivariate Analysis**  **OR (95% CI)** |
| Age | | 0.97 (0.94–1.00) | NS |
| Gender | | NS | NS |
| IC diagnosis | | 5.80 (3.02–12.06) | 3.66 (1.29–11.42) |
| FAP diagnosis | | 0.58 (0.42–0.80) | NS |
| Mother being the respondent of the survey  Parental education | | 1.89 (1.13–3.25)  NS | 5.29 (1.94–17.24)  NS |
| High household income family (>€70.000) | | 1.31 (0.91–1.89) | 2.23 (1.19–4.26) |
| No perceived effect of treatment by pediatrician | | 0.88 (0.48–1.62) | NS |
| Gastrointestinal symptoms >3 years | | 1.32 (0.95–1.84) | 2.15 (1.10–4.27) |
| *NS: not significant*  ** If patients reported having used hypnotherapy for FAP, we removed this from the analysis in this manuscript as hypnotherapy is not considered CAM and is prescribed according to current clinical guidelines in this cohort.* | | | |

**Appendix 3**

| Table 3 | CAM therapies used by patients per diagnosis | | | | | |
| --- | --- | --- | --- | --- | --- | --- |
|  | | No. of users (%)* | | | | |
| CAM therapies/ Diagnosis | | **FC (*N* = 299)** | **FAP (*N* = 266)** | **IC (*N* = 52)** | **GERD (*N* = 39)** | **Total (*N* = 655)**  ******* |
| Manual therapies | | 82 (27.4) | 44 (16.5) | 37 (71.2) | 15 (38.5) | 178 (27.2) |
| Homeopathy | | 35 (11.7) | 32 (12.0) | 6 (11.5) | 7 (17.9) | 80 (12.2) |
| Natural remedies | | 31 (10.4) | 30 (11.3) | 3 (5.8) | 0 (0) | 64 (9.8) |
| Hypnotherapy | | 17 (5.7) | --** | 0 (0) | 5 (12.8) | 23 (3.5) |
| Others | | 10 (3.3) | 11 (4.1) | 2 (3.8) | 0 (0) | 23 (3.5) |
| Other Chinese medicine | | 7 (2.3) | 11 (4.1) | 1 (1.9) | 0 (0) | 19 (2.9) |
| Acupuncture | | 7 (2.3) | 8 (3.0) | 1 (1.9) | 0 (0) | 16 (2.4) |
| Magnetism/ paranormal therapies | | 8 (2.7) | 5 (1.9) | 0 (0) | 1 (2.6) | 14 (2.2) |
| Anthroposophic medicine | | 4 (1.3) | 4 (1.3) | 0 (0) | 2 (5.1) | 10 (1.5) |
| Never used any CAM | | 176 (58.9) | 175 (65.8) | 11 (21.2) | 17 (43.6) | 378 (57.7) |
| Used CAM | | 123 (41.1) | 91 (34.2) | 41 (78.8) | 22 (56.4) | 277 (42.3) |
| Used one CAM | | 70 (23.4) | 54 (20.3) | 35 (67.3) | 16 (41.0) | 175 (26.7) |
| Used more than one CAM | | 53 (17.7) | 37 (13.9) | 6 (11.5) | 6 (15.4) | 102 (15.6) |
| **Of the respondents*  *** 55 of 266 FAP patients (20.7%) used hypnotherapy, which is not classified as CAM for FAP. Hypnotherapy for FAP is not included, while it is included for other conditions.*  **** There were 655 patients with FC, FAP, IC, and/or GERD who provided information about CAM use. One patient had two Rome IV diagnoses (FC and FAP), this patient reported hypnotherapy as the only CAM the patient had used. As hypnotherapy for FAP is not classified as CAM in our analysis, this case was excluded from both the FC hypnotherapy and overall CAM prevalence calculations to avoid overestimation.* | | | | | | |

**Appendix 4**

Extra overview **including hypnotherapy for FAP**

| Table 4A | Recommended CAM therapies by pediatricians, including hypnotherapy for FAP patients | | |
| --- | --- | --- | --- |
|  | | **No. of recommenders (%)** | |
| CAM therapies/ Diagnosis | | **FAP (*N* = 73)** | **Total (*N* = 76)** |
| Hypnotherapy | | **72 (98.6)** | **73 (96.1)** |
| Manual therapies | | 3 (4.1) | 22 (28.9) |
| Others | | 3 (4.1) | 8 (10.5) |
| Homeopathy | | 4 (5.5) | 7 (9.2) |
| Natural remedies | | 2 (2.7) | 6 (7.9) |
| Other Chinese medicine | | 1 (1.4) | 2 (2.6) |
| Acupuncture | | 1 (1.4) | 2 (2.6) |
| Anthroposophic medicine | | 0 (0) | 1 (1.3) |
| Magnetism/ paranormal therapies | | 0 (0) | 0 (0) |
| Never recommended any CAM | | 1 (1.4) | 2 (2.6) |
| Recommended CAM | | 72 (98.6) | 74 (97.4) |
| Recommended one CAM | | 61 (13.7) | 36 (47.4) |
| Recommended more than one CAM | | 11 (1.4) | 38 (50.0) |

| Table 4B | CAM therapies used by FAP patients | | |
| --- | --- | --- | --- |
|  | | No. of users (%) | |
| CAM therapies/ Diagnosis | | **FAP (*N* = 266)** | **Total (*N* = 655)** |
| Manual therapies | | 44 (16.5) | 178 (27.2) |
| Homeopathy | | 32 (12.0) | 80 (12.2) |
| Hypnotherapy | | **55 (20.7)** | **78 (11.9)** |
| Natural remedies | | 30 (11.3) | 64 (9.8) |
| Others | | 11 (4.1) | 23 (3.5) |
| Other Chinese medicine | | 11 (4.1) | 19 (2.9) |
| Acupuncture | | 8 (3.0) | 16 (2.4) |
| Magnetism/ paranormal therapies | | 5 (1.9) | 14 (2.2) |
| Anthroposophic medicine | | 4 (1.3) | 10 (1.5) |

**Appendix 5. Perceived effectiveness of CAM**

| **Table 5A** | **Perceived effectiveness of each CAM therapy** | | | | | | | | | | |
| --- | --- | --- | --- | --- | --- | --- | --- | --- | --- | --- | --- |
| Perceived efficacy of CAM* | | Hypnotherapy**  (*N* = 22) | Acupuncture  (*N* = 16) | Other Chinese medicine  (*N* = 19) | Natural remedies  (*N* = 64) | Anthroposophic medicine  (*N* = 10) | Manual therapies  (*N* = 178) | Magnetism/ paranormal therapies  (*N* = 14) | Homeopathy (*N* = 80) | Others  (*N* = 23) | All CAM therapies (*N* = 426), *N* (%) |
| Very effective | | 1 (4.5) | 2 (12.5) | 2 (10.5) | 6 (9.4) | 1 (10.0) | 31 (17.4) | 0 (0.0) | 4 (5.0) | 3 (13.0) | 50 (11.7) |
| Somewhat effective | | 4 (18.2) | 7 (43.8) | 9 (47.4) | 27 (42.2) | 6 (60.0) | 71 (39.9) | 5 (35.7) | 34 (42.5) | 9 (39.1) | 172 (40.4) |
| Not effective | | 14 (63.6) | 4 (25.0) | 6 (31.6) | 20 (31.3) | 1 (10.0) | 64 (36.0) | 9 (64.3) | 32 (40.0) | 6 (26.1) | 156 (36.6) |
| Unknown | | 3 (13.6) | 3 (18.8) | 2 (10.5) | 11 (17.2) | 2 (20.0) | 12 (6.7) | 0 (0.0) | 10 (12.5) | 5 (21.7) | 48 (11.3) |

| **Table 5B** | **Perceived effectiveness of hypnotherapy including for FAP patients** | |
| --- | --- | --- |
| Perceived efficacy of CAM | Hypnotherapy  (*N* = 77) | All CAM therapies (*N* = 481), *N* (%) |
| Very effective | 3 (0.4) | 52 (10.8) |
| Somewhat effective | 18 (2.7) | 186 (38.7) |
| Not effective | 40 (51.9) | 182 (37.8) |
| Unknown | 16 (2.4) | 61 (12.7) |

**Reasons for choosing CAM**

Parents were asked to indicate which arguments played or would play a role in their decision to use alternative therapies for their child. They could select multiple reasons from a list of standard options (e.g., recommendation by a healthcare provider, concerns about medication side effects, preference for natural treatments, preference to use fewer medications, disappointment with conventional treatment, feeling of having more control over their child’s illness, need to do something for their child, wish to improve their child’s general resistance and condition, advice from family and friends, personal positive experience with CAM, information seen on the internet or television, or no reason to consider CAM). Parents were also given the opportunity to provide additional reasons in an open-ended response.

Among parents reporting CAM use for their child, the main reasons for choosing CAM therapies were: wishing to cure the condition (82%), having the feeling of more control or being able to do something for their child’s illness and health (46.2%), preference to use less medication (42.2%), recommendation by friends and/or family (37.2%), recommendation by a general practitioner (36.5%), improving general health (35.7%), disappointment with current treatment by the pediatrician (31%), preference for a more ‘natural’ treatment (27.1%), and experiencing fewer side effects from medicines (24.6%). Less than 10% of parents reported choosing CAM because it aligns more closely with their lifestyle (8.3%) or due to information seen on the internet or television (6.9%).

Among parents who provided open-text responses, thematically clustered answers included: long waitlists for referral to a pediatrician (n=7, 1.1%); using CAM as an interim step to determine which specialist in conventional care was needed (n=1, 0.4%); and a wish to combine conventional and alternative treatments (n=12, 4.3%). One parent reported that, after trying all available CAM therapies without effect, they would not consider CAM again (n=1, 0.4%).

Additionally, amongst parents reporting *no* CAM use for their child, 71.2% reported considering using CAM if it were recommended by their treating pediatrician, 36.5% would considering using CAM if it were recommended by their general practitioner, 28.8% would consider CAM use to improve general health of their child, 26.2% would consider CAM use as a proactive stance on their child’s health, 14.8% would consider CAM use if they were disappointed in conventional treatment results, and 14.3% of parents reported they did believe CAM fit their lifestyle, and 13.8% reported having positive personal experiences with CAM use. Only 15.9% of the parents who reported no CAM use for their child at time of survey stated that they had no reason to ever consider CAM.

**Overige door ouders opgegeven redenen voor CAM-gebruik, per CAM-type**

| **CAM-type** | **Thema/reden** |
| --- | --- |
| **Hypnotherapie** | **Advies/verwijzing arts**: verwijzing door kinderarts, advies kinderarts, aanbevolen, net begonnen op aanraden van kinderarts **Symptoomverlichting/klachten**: buikpijn, minder gespannen zijn/minder paniek, verlichting bij PDS, ontspanning waardoor minder pijn, behandeling van mogelijke stress, meer zelfvertrouwen/verwerking, geleerd anders ademen/rustig blijven, omgang met de klachten, manier om om te gaan met de klachten, de vicieuze cirkel proberen te doorbreken, voor slikken medicatie **Proberen/laatste redmiddel**: proberen waard, proberen of het verbetering zou geven **Geen alternatief helpt**: geen alternatief helpt **Overig**: omdat ze de oorzaak niet kunnen vinden, beter ademen |
| **Chinese geneeskunde** | Meer ontspanning in het bekken en algehele lijfje van onze baby. Op zoek naar een oplossing, nog niet gevonden |
| **Natuurgeneeskunde** | Proberen de klachten te verlichten, darmen legen, incontinentie/obstipatie, oorzaak achterhalen en proberen te verhelpen, het ging er hierbij vooral om dat hij meer "los" ging laten (hij hield zijn ontlasting tegen...), omdat we alles wilden proberen om haar van obstipatie af te helpen (eigen kinderarts in Enschede deed niks, zat tussen de oren...), wij vinden het belangrijk om ook breder te kijken dan alleen reguliere geneeskunde (orthomoleculair therapeut) |
| **Manuele therapie** | **Advies/verwijzing arts**: op advies van iemand, doorverwijzing mdl arts, door verwezen door arts, op advies van lactatiekundige **Symptoomverlichting/klachten**: spanningshoofdpijn, veel krampen, last van nek met draaien, nek/bovenkant hoofd/heup, check up, verminderen spanning, schijnt bij huilbaby te helpen, adviezen, last van wervel nekje, hoge spanning in lijfje dochter, als baby 4 behandelingen voor buikklachten, als baby last van buik/ter voorkoming van reflux achtige klachten zoals bij broer, veel huilen vanaf maand 2 (dachten dat osteopathie kon helpen), huilbaby (dat werd veel minder) en moeilijke ontlasting (weinig verbetering), huilen verminderen, geven van rust, vermindering velen huilen, enorm gespannen baby (na ieder bezoek aan osteopaat veel verbetering), reflux werd minder, baby kon vrijer bewegen, overmatige huilen nam af, vermindering huilklachten baby, in medische circuit werden klachten gebagatelliseerd, veel huilen/onrustig/gespannen **Proberen/laatste redmiddel**: proberen, we hebben van alles geprobeerd om haar van haar klachten af te helpen, poging om te bekijken of deze therapie verduidelijking zou geven van de klachten, proberen of onze zoon zo beter zijn aandrang kon voelen, alles willen proberen om kind te helpen, we werden toen de tijd niet gehoord in het ziekenhuis, was nog voordat we naar het ziekenhuis gingen, eerdere ervaring, we wilde proberen of de osteopaat iets zou merken aan onze zoon en of hij tips had m.b.t. (verborgen) reflux en excessief huilen, we hebben 2 bezoeken gehad, slechts intake gehad ter informatie (geen behandeling gestart) **Specifiek functioneel doel**: trainen van sluitspier, verborgen reflex, leren om te staan en balans te houden, diëten uitproberen/manier van verschonen op school, inzicht in spanning die ze opbouwt in haar lichaam en oefeningen leren om dat in de dagelijkse praktijk te ontspannen, omgang met klachten en te handelen bij klachten |
| **Magnetisme/paranormaal** | Hopen op verbetering, darmen legen, beeld krijgen wat er energetisch speelt bij mijn dochter, door verwezen door arts, verbeteren gezondheid ook mentaal gezien, vol hoofd |
| **Alternatief (overig)** | Omdat niks anders helpt, craniosacraal theorie over werking schedelbotjes en bekken na een te snelle bevalling (theorie leek overeen te komen met gebeurtenissen rondom de bevalling, wilden het proberen; zoon had minder last van nekje na behandeling, bewoog soepeler, gunstig voor obstipatieprobleem), wegnemen blokkade verstopping, stevige ontlasting, op zoek naar de bron van de klachten (waar in het lichaam komt de spanning/buikpijn/kramp vandaan?) |

**Appendix 6**

| Table 6A | Scores on the BMQ General (*N* = 625) and BMQ Specific (*N* = 347) | | | | | |
| --- | --- | --- | --- | --- | --- | --- |
| Scales of the questionnaire | | Range of score | Mean Score | S.D. | % scoring above the scale midpoint | |
| Harm* | | 4 to 20 | 9.8 | 2.4 | | 10.7 |
| Overuse* | | 4 to 20 | 11.4 | 2.8 | | 33.1 |
| BMQ General (Harm – Overuse)*** | | -16 to 16 | -1.6 | 2.4 | | 15.0 |
| Necessity** | | 5 to 25 | 15.3 | 3.9 | | 50.1 |
| Concern** | | 5 to 25 | 12.5 | 3.3 | | 17.9 |
| BMQ Specific (Necessity – Concern)**** | | -20 to 20 | 2.7 | 3.5 | | 73.8 |
| **Midpoint: 12 (range 4 – 20)*  ***Midpoint: 15 (range 5 – 25)*  ****Midpoint: 0 (range -16 – 16)*  *****Midpoint: 0 (range -20 – 20)* (59-61) | | | | | | |

| Table 6B | Scale means and standard deviations for BMQ scales for CAM users and non-CAM users | | | | | |
| --- | --- | --- | --- | --- | --- | --- |
| Scale | | CAM users | Non- CAM users | *t* test | *p* | 95% CI |
| Harm | | (*N* = 265) | (*N* = 360) |  |  |  |
| Mean | | 10.0 | 9.6 | -2.805 | 0.037 | [-0.797, -0.024] |
| SD | | 2.4 | 2.4 |  |  |  |
| Overuse | | (*N* = 265) | (*N* = 360) |  |  |  |
| Mean | | 11.8 | 11.0 | -3.167 | 0.002 | [-1.160,-0.267] |
| SD | | 2.9 | 2.7 |  |  |  |
| BMQ General (Harm - Overuse) | | (*N* = 265) | (*N* = 360) |  |  |  |
| Mean | | -1.8 | -1.4 | 1.601 | 0.110 | [-0.069, 0.680] |
| SD | | 2.4 | 2.3 |  |  |  |
| Necessity* | | (*N* = 142) | (*N* = 205) |  |  |  |
| Mean | | 15.8 | 14.9 | -2.155 | 0.032 | [-1.727,-0.079] |
| SD | | 3.9 | 3.8 |  |  |  |
| Concern* | | (*N* = 142) | (*N* = 205) |  |  |  |
| Mean | | 13.0 | 12.2 | -2.453 | 0.015 | [-1.570,-0.173] |
| SD | | 3.0 | 3.4 |  |  |  |
| BMQ Specific (Necessity - Concern)* | | (*N* = 142) | (*N* = 205) |  |  |  |
| Mean | | 2.8 | 2.7 | -0.082 | 0.935 | [-0.789, 0.726] |
| SD | | 3.4 | 3.6 |  |  |  |
| ** BMQ specific questions were only applicable in case of current medicine use. In this patient population only 347 patients had currently used medication during this study.* | | | | | | |

| Table 6C | BMQ General for subgroups never used CAM (*N* = 360) and ever used CAM (*N* = 265) | | | | | | |
| --- | --- | --- | --- | --- | --- | --- | --- |
|  | | | Prevalence, *n* (%) | | | | |
|  |  |  | Strongly disagree | Disagree | Neutral | Agree | Strongly agree |
| Harm | | | | | | | |
| 1. Most medicines are addictive. | | Never  used CAM | 21 (5.8) | 178 (49.4) | 114 (31.7) | 40 (11.1) | 7 (1.9) |
|  |  | Ever used CAM | 13 (4.9) | 120 (45.3) | 89 (33.6) | 38 (14.3) | 5 (1.9) |
| 1. Natural remedies are safer than medicines. | | Never  used CAM | 15 (4.2) | 124 (34.4) | 157 (43.6) | 57 (15.8) | 7 (1.9) |
|  |  | Ever  used CAM | 9 (3.4) | 73 (27.5) | 108 (40.8) | 60 (22.6) | 15 (5.7) |
| 1. Medicines do more harm than good. | | Never  used CAM | 38 (10.6) | 200 (55.6) | 96 (26.7) | 22 (6.1) | 4 (1.1) |
|  |  | Ever  used CAM | 21 (7.9) | 144 (54.3) | 80 (30.2) | 16 (6.0) | 4 (1.5) |
| 1. All medicines are poison. | | Never  used CAM | 101 (28.1) | 183 (50.8) | 60 (16.7) | 13 (3.6) | 3 (0.8) |
|  |  | Ever  used CAM | 76 (28.7) | 132 (49.8) | 42 (15.8) | 10 (3.8) | 5 (1.9) |
| Overuse | |  |  |  |  |  |  |
| 1. Doctors use too many medicines. | | Never used CAM | 18 (5.0) | 171 (47.5) | 94 (26.1) | 69 (19.2) | 8 (2.2) |
|  |  | Ever used CAM | 10 (3.8) | 105 (39.6) | 64 (24.2) | 75 (28.3) | 11 (4.2) |
| 1. People who take medicines should stop their treatment for a while every now and again. | | Never  used CAM | 15 (4.2) | 108 (30.0) | 129 (35.8) | 92 (25.6) | 16 (4.4) |
|  |  | Ever  used CAM | 12 (4.5) | 72 (27.2) | 92 (34.7) | 80 (30.2) | 9 (3.4) |
| 1. Docters place too much trust in medicines. | | Never  used CAM | 26 (7.2) | 153 (42.5) | 117 (32.5) | 61 (16.9) | 3 (0.8) |
|  |  | Ever  used CAM | 10 (3.8) | 96 (36.2) | 85 (32.1) | 66 (24.9) | 8 (3.0) |
| 1. If doctors had more time with patients, they would prescribe fewer medicines. | | Never  used CAM | 22 (6.1) | 131 (36.4) | 115 (31.9) | 77 (21.4) | 15 (4.2) |
|  |  | Ever  used CAM | 17 (6.4) | 86 (32.5) | 66 (24.9) | 74 (27.9) | 22 (8.3) |

| Table 6D | BMQ Specific for subgroups never used CAM (*N* = 205) and ever used CAM (*N* = 143) | | | | | | |
| --- | --- | --- | --- | --- | --- | --- | --- |
|  | | | Prevalence, *n* (%) | | | | |
|  |  |  | Strongly disagree | Disagree | Neutral | Agree | Strongly agree |
| Necessity | | | | | | | |
| 1. My child’s health, at present, depends on his/her medicines. | | Never  used CAM | 17 (6.4) | 86 (32.5) | 66 (24.9) | 74 (27.9) | 22 (8.3) |
|  |  | Ever  used CAM | 7 (4.9) | 33 (23.1) | 8 (5.6) | 73 (51.0) | 22 (15.4) |
| 1. The fact that my child having to take medicines worries me. | | Never  used CAM | 11 (5.4) | 111 (54.1) | 16 (7.8) | 16 (7.8) | 13 (6.3) |
|  |  | Ever  used CAM | 6 (4.2) | 51 (35.7) | 9 (6.3) | 65 (45.5) | 12 (8.4) |
| 1. My child’s life would be impossible without his/her medicines. | | Never  used CAM | 29 (14.1) | 83 (40.5) | 27 (13.2) | 58 (28.3) | 8 (3.9) |
|  |  | Ever  used CAM | 16 (11.3) | 54 (38.0) | 27 (19.0) | 33 (23.2) | 12 (8.5) |
| 1. Without the medicines, my child would be very ill. | | Never  used CAM | 23 (11.2) | 80 (39.0) | 25 (12.2) | 67 (32.7) | 10 (4.9) |
|  |  | Ever  used CAM | 13 (9.2) | 44 (31.0) | 27 (19.0) | 46 (32.4) | 12 (8.5) |
| 1. My child’s medicines protect his/her from becoming worse. | | Never  used CAM | 13 (6.3) | 40 (19.5) | 46 (22.4) | 91 (44.4) | 15 (7.3) |
|  |  | Ever  used CAM | 7 (4.9) | 25 (17.6) | 38 (26.8) | 59 (41.5) | 13 (9.2) |
| Concern | | | | | | | |
| 1. I sometimes worry about the long-term effects of my child’s medicines. | | Never  used CAM | 18 (8.8) | 78 (38.0) | 25 (12.2) | 73 (35.6) | 11 (5.4) |
|  |  | Ever  used CAM | 6 (4.2) | 37 (26.1) | 9 (6.3) | 74 (52.1) | 16 (5.8) |
| 1. My child’s medicines are a mystery to me. | | Never  used CAM | 51 (24.9) | 122 (59.5) | 16 (7.8) | 12 (5.9) | 4 (2.0) |
|  |  | Ever  used CAM | 39 (27.5) | 85 (59.9) | 12 (8.5) | 5 (3.5) | 1 (0.7) |
| 1. My child’s health in the future will depend on his or her medication. | | Never  used CAM | 26 (12.7) | 92 (44.9) | 47 (22.9) | 32 (15.6) | 8 (3.9) |
|  |  | Ever  used CAM | 15 (10.6) | 52 (36.6) | 44 (31.0) | 26 (18.3) | 5 (3.5) |
| 1. My child’s medicines disrupt his/her life. | | Never  used CAM | 47 (22.9) | 120 (58.5) | 18 (8.8) | 17 (8.3) | 3 (1.5) |
|  |  | Ever  used CAM | 27 (19.0) | 86 (60.6) | 12 (8.5) | 13 (9.2) | 4 (2.8) |
| 1. I sometimes worry about my child becoming too dependent on his/her medicines. | | Never  used CAM | 27 (13.2) | 97 (47.3) | 9 (4.4) | 63 (30.7) | 9 (4.4) |
|  |  | Ever  used CAM | 14 (9.9) | 54 (38.0) | 13 (9.2) | 54 (38.0) | 7 (4.9) |

Overall, 10.7% of parents perceived medicines as harmful, 33.1% believed doctors overprescribe, 50.1% strongly endorsed the necessity of their child’s medicines, and 17.9% expressed concerns about potential side effects. CAM users scored slightly higher than non-users on harm, overuse, necessity, and concern BMQ subscales, indicating greater skepticism about medicines and concerns regarding their use, but mean differences were small (harm: 10.0 vs 9.6; overuse: 11.8 vs 11.0). No significant differences were observed for the BMQ General (harm–overuse) or Specific (necessity–concern) difference scores, suggesting that overall similar beliefs about medicines between groups. Among respondents currently using medication, 66.4% of CAM-users agreed that their child’s health depends on medicines (vs 36.2% of non-users), and 53.9% of CAM users worried about their child taking medicines (vs 14.1% of non-users).

**Appendix 7.**

| Table 7A | Pediatricians’ demographics | | | | | | | |  |
| --- | --- | --- | --- | --- | --- | --- | --- | --- | --- |
| Characteristic | | | | Prevalence, *n* (%) | | | | |  |
| Pediatrician specialization (*N* = 76) | | | |  | | | | |  |
| Pediatrician (general) | | | | 65 (85.5) | | | | |  |
| Pediatric gastroenterologist | | | | 5 (6.6) | | | | |  |
| Neonatologist | | | | 3 (4.0) | | | | |  |
| Other* | | | | 3 (3.9) | | | | |  |
| Type of Hospital (*N* = 76) | | | |  | | | | |  |
| General Hospital | | | | 70 (92.1) | | | | |  |
| Academic Hospital | | | | 6 (7.9) | | | | |  |
| Work experience (*N* = 76) | | | |  | | | | |  |
| 0-5 years | | | | 19 (25) | | | | |  |
| 6-10 years | | | | 13 (17.1) | | | | |  |
| 11-15 years | | | | 5 (6.6) | | | | |  |
| >15 years | | | | 39 (51.3) | | | | |  |
| Ethnicity (*N* = 76) | | | |  | | | | |  |
| Caucasian | | | | 73 (96.1) | | | | |  |
| Not specified | | | | 2 (2.6) | | | | |  |
| Mixed Caucasian | | | | 1 (1.3) | | | | |  |
| Others, namely** | | | | 0 (0) | | | | |  |
| ** 1 pediatric pulmonologist, 1 pediatric neurologist & 1 pediatrician hereditary and congenital disorders.*  *** Afro-Caribbean, Arabic, African, Indian/Pakistani/Bengalese, Latin American & Asian.* | | | | | | | | |  |
| Table 7B | | **Recommended CAM therapies per diagnosis by pediatricians** | | | | | | | |
|  | | | No. of recommenders (%)* | | | | | | |
| CAM therapies/ Diagnosis | | | **FC (*N* = 73)** | | **FAP (*N* = 73)** | **IC (*N* = 69)** | **GERD (*N* = 71)** | **Total (*N* = 76)***** | |
| Hypnotherapy | | | 20 (27.4) | | --** | 0 (0) | 9 (12.7) | 29 (38.2) | |
| Manual therapies | | | 1 (1.4) | | 3 (4.1) | 15 (21.7) | 3 (4.2) | 22 (28.9) | |
| Other**** | | | 2 (2.7) | | 3 (4.1) | 2 (2.9) | 1 (1.4) | 8 (10.5) | |
| Homeopathy | | | 3 (4.1) | | 4 (5.5) | 0 (0) | 0 (0) | 7 (9.2) | |
| Natural remedies | | | 2 (2.7) | | 2 (2.7) | 2 (2.9) | 0 (0) | 6 (7.9) | |
| Other Chinese medicine | | | 1 (1.4) | | 1 (1.4) | 0 (0) | 0 (0) | 2 (2.6) | |
| Acupuncture | | | 1 (1.4) | | 1 (1.4) | 0 (0) | 0 (0) | 2 (2.6) | |
| Anthroposophic medicine | | | 1 (1.4) | | 0 (0) | 0 (0) | 0 (0) | 1 (1.3) | |
| Magnetism/ paranormal therapies | | | 0 (0) | | 0 (0) | 0 (0) | 0 (0) | 0 (0) | |
| Never recommended any CAM | | | 49 (67.1) | | 62 (84.9) | 51 (73.9) | 58 (81.7) | 36 (47.4) | |
| Recommended CAM | | | 24 (32.9) | | 11 (15.1) | 18 (26.1) | 13 (18.3) | 40 (52.6) | |
| one CAM | | | 18 (24.7) | | 10 (13.7) | 16 (23.2) | 13 (18.3) | 21 (27.6) | |
| more than one CAM | | | 6 (8.2) | | 1 (1.4) | 2 (2.9) | 0 (0) | 19 (25.0) | |
| **Of the respondents*  *** 72 of the 73 pediatricians (98.6%) recommended hypnotherapy, which is not classified as CAM for FAP. Hypnotherapy for FAP is not included, while it is included for other conditions.*  **** 76 pediatricians responded to the survey at least partly. Not all pediatricians reported treating all four diagnoses, which is why the total number per diagnosis is lower than the total number of pediatricians reporting CAM use.*  *****Other therapies: dietician, medical taping, mindfulness, peppermint oil, yoga, simethicone and physiotherapy.* | | | | | | | | | |
